# Silently Proliferating: Cancer

**DOI:** 10.5152/eurasianjmed.2022.22325

**Published:** 2022-12-01

**Authors:** Gulay Ipek Coban, Aytap Dincer, Asena Kose

**Affiliations:** 1 Faculty of Nursing, Department of Fundamental of Nursing, Ataturk University, Erzurum, Turkey; 2 Department of Fundamental of Nursing, Ataturk University, Erzurum, Turkey

**Keywords:** Burden caregiver, cancer, quality of life, wellness

## Abstract

Cancer is a health problem that has existed for years and frequently affects tissues and organs. Although some standards have been set for its treatment, different treatments and approaches are used for each type of cancer. The treatment process and having to live with cancer drag the individual to a difficult process that affects the individual negatively. Problems such as the severe course of the disease, taking longer than expected treatment, and having many side effects reduce the individual's physical performance and activities of daily living. Therefore, this situation reduces the quality of life and causes the general well-being of the individual to be negatively affected at the psycho-social level. In order to cope with the problems that cancer patients experience, it is important to support them physically, mentally, emotionally, socio-culturally, and spiritually. The decrease in the quality of life of the caregiver also affects the quality of care and therefore the quality of life of the patient. For this reason, it is necessary to determine how patients and caregivers perceive the disease, reduce their psychological anxiety and pain, as well as the factors affecting this perception, and ensure their compliance with treatment.

## Introduction

Cancer has existed and is known throughout history, and it is a common problem in animals and humans. The earliest known records of cancer date back to 3000 BC. Cancer is the uncontrolled accumulation, division, and proliferation of cells in an organism. It affects a single organ or distant organs. Although some standards have been set for its treatment, different treatments and approaches are used for each type of cancer. In addition to cancer treatment, identifying the factors that cause cancer, preventing cancer before it occurs, and providing social and psychological support to patients and their relatives make cancer an extremely complicated problem. To solve these problems, oncology uses different branches of medicine. In cancer treatment, biological therapies, such as chemotherapy, radiotherapy, hormone therapy, surgery, immunotherapy, and gene therapy, are used alone or in combination.^1,2^

### Prevalence and Significance of Cancer in Turkey and Around the World

Cancer is one of the most common causes of death in the world. Receiving a cancer diagnosis is a very stressful experience. Significant negative physical, economic, and psychosocial consequences occur for individuals with cancer. Patients with advanced cancer experience difficulty living in the face of death. Psychological problems are frequently observed in the clinical course of this disease. Patients face problems such as unresolved problems, fear of death, pain, and separation from family.^3^ According to the data published by the World Health Organization (WHO), the global cancer burden in the last 30 years has doubled. The cellular immune system plays an important role in cancer and inflammation and the inflammatory response. Inflammation causes cancer to progress, increases the risk of tumors, and affects the stages of all cancer stages.^4^

There are more than 100 types of cancer known to affect the human body. Lung cancer, one of them, is in the first place of cancer-related deaths in women and men worldwide. Lung cancer patients present with a wide variety of severe physical and psychological symptoms related to the side effects of drugs and the natural course of the disease. The main treatments for lung cancer are radiotherapy and chemotherapy. Treatment is aimed at improving, controlling, or relieving symptoms. However, such treatments can affect patients physically, emotionally, and socially and increase their symptoms.^5-9^ On the other hand, chemotherapy and radiotherapy in patients with lung cancer provide good results in very few people. For this reason, research on cancer has generally pointed to the relationship between genetic change and the clinical manifestation of lung cancer. Today, it is thought that different treatment options, especially gene therapy and immunotherapy, are needed for lung cancer.^10-12^

Breast cancer is the second most common cancer type in the world after lung cancer. It is a very important health problem worldwide. Its incidence is increasing gradually.^13,14^ About 1.38 million women are diagnosed with breast cancer each year. Approximately 458 000 women lose their lives because of this. Breast cancer affects approximately 15 000 women each year in Turkey. Breast cancer accounts for 20%-25% of all cancer cases among women.^15,16^ Age is one of the most important risk factors for breast cancer. As average life expectancy increases, the incidence of breast cancer also increases.^14,17-19^ More than 30% of women with breast cancer are over the age of 70. As in chronic diseases, screening programs are very important to raise awareness in cancer patients. Individuals' perceptions of obstacles, if any, should be learned and strategies should be developed for this.^20-22^ One of the most effective methods in the fight against cancer is cancer screening programs. If breast cancer is detected at an early stage with screening, it can be completely cured. Turkey complies with the recommendations of the WHO on cancer screening. Women voluntarily participate in these screening programs. Women should be encouraged to perform monthly breast self-exams, and clinical breast exams should be performed annually. Women between the ages of 40 and 69 are recommended to have mammography every 2 years.^16,23,24^ In addition, education and counselling programs should be carried out before, during, and after the treatment by raising awareness about the treatment and diagnosis process of breast cancer.^24-26^ In recent years, there have been important developments in breast cancer management, including adjuvant drug therapy and surgical procedures. With these advances, treatment-related symptoms are still more common. Patients try to reduce treatment-related symptoms using alternative and complementary therapies. They also aim to improve their quality of life. One of the alternative and complementary treatments frequently used in breast cancer patients is energy therapy.^17,27-29^ In addition, breast cancer, which is common among women, is a disease with many psychological and social aspects because it arises in an organ that often represents sexuality and femininity. Women often go through a process that includes physical, psychological, and social changes after breast cancer. The most important point in this process is the positive adaptation of patients to possible changes. The body image of a woman may be adversely affected, especially if mastectomy develops as a result. Therefore, health professionals should consider marital adjustment and hopelessness levels when treating women who have undergone mastectomy.^30,31^

Finally, gastric cancer is the third most common type of cancer after lung and breast cancers, which causes death worldwide. Therefore, it is a global health problem for all nations.^32-35^ Especially *Helicobacter pylori* infections, consumption of very salty foods, and environmental exposures are risk factors for gastric cancer.^7^ It is an important public health problem in Turkey, especially in Eastern Anatolia region.^36^ However, esophageal cancers constitute 5% of all cancers. In Turkey, this rate is 16% in the Eastern Anatolia region.^37,38^

### Cancer Patients in Many Ways

Cancer is an important health problem in developed or developing countries, with advanced treatment methods to increase the life expectancy and quality of life of patients. Although efforts in this direction have been largely met, it has been reported that patients experience problems in many aspects from the moment they are diagnosed, during the cure process, after, and in the terminal period.^39^ These questions affect the well-being and physical fitness of the patients, cause delay or limitation of the treatment, and affect the prognosis; they are situations in which the individual experiences physical and emotional suffering by disrupting their physical, mental, and social functions.^40^

It is important for cancer patients to be supported physically, mentally, emotionally, socioculturally, and spiritually so that they can cope with the problems they experience.^41^ Therefore, it is significant to determine how cancer patients perceive their disease, to reduce their psychological anxiety and pain, to ensure their compliance with treatment, and to increase their quality of life, as well as the factors that affect this perception. To achieve this, patients should be helped to express themselves, and those who have difficulties in this regard should be supported.^42^ In particular, strengthening the spirituality of patients is of great importance, and this is possible with individual initiatives. Spirituality, which means finding inner peace and feeling good through non-physical means, such as seeking meaning and purpose in life, praying, and meditating, is critically important for coping with diseases such as cancer and supporting well-being, treatment, and recovery.^43^ Similarly, hope is an important coping mechanism for overcoming the existential crisis caused by cancer. It has been reported that hope, which is a multidimensional trigger for the future, reduces psychological distress and increases quality of life and satisfaction in cancer patients, while mental well-being reduces depression and hopelessness.^44-46^ In addition, patients seek social support, which is a problem-focused coping strategy, in the early stages of their illness. However, as the duration of the illness increases, perceived social support also decreases.^47^ For this reason, it should not be forgotten that terminal cancer patients in particular experience intense loneliness and should be supported. It is of great importance to increase the social support of patients in this process and to increase their mental well-being.^48^

The symptoms that cancer patients have to deal with and the side effects that develop due to the treatment they receive are important points for consideration because they may restrict the individual’s daily life activities and social interactions. In this case, it is necessary to develop care strategies for the problems experienced by the individual. For example, in prostate cancer patients receiving radiotherapy, urinary and fecal incontinence may develop after radiotherapy. Kegel exercises have been reported to have positive effects on reducing side effects in these patients.^49^ In this respect, complementary therapies come to the fore. For example, reflexology, a complementary therapy, has positive effects on the general health status of breast cancer patients receiving chemotherapy and increases functionality. Reflexology improves the quality of life in breast cancer patients; it has positive effects on nausea, vomiting, retching, and fatigue. It has been reported that it has potential benefits by significantly reducing chemotherapy and disease-related symptoms.^50,51^

### Caring Burdens of Caregivers of Cancer Patients

Illness is unexpected for individuals and their families, and families are undesirably affected by situations in which patients experience a significant inability to fulfill their self-care needs. Caregivers who cannot be chosen or planned pose many difficulties for the caregiver and the patient.^52^ Because of the nature of the disease, the symptoms experienced by individuals cause caregivers to experience stress, sleep disorders, anxiety, and burnout, as well as a significant care burden for caregiver. Providing the necessary support to the person in need of care, negative experiences, difficulties, all kinds of stress, and reactions to the care of the individual are called care burdens.^53^ In addition, when the cultural structure of Turkish society is examined, family members are generally responsible for the care of patients, and this responsibility brings an inevitable burden on family members.^54,55^

The patients with cancer can cause to lose control over the life of caregivers to lose control over their lives; It is a chronic disease that negatively affects their social, work, family, and marital life, health, and quality of life.^39^ Also, considering the incidence of cancer, which is the second highest cause of death among adults, more and more families in Turkey have to live with and care for relatives with cancer. Family caregivers, who play a central role in managing all aspects of patient care, cause caregivers to be affected by this structure, since the family structure has an extremely important place in traditional Turkish culture.^56^ In addition, the fact that the home care system is not well-established causes informal care to be more common than formal care. The responsibility for care falls on the spouse, children, relatives, or friends of the patient.^57^

Family caregivers may have to cope with various physical, social, and economic problems in the process of caring for cancer patients who experience high levels of pain, fatigue, nausea, and depression.^50,58-60^ A decrease in the quality of life of the caregiver affects the quality of care and therefore, the quality of life of the patient. Research on family caregivers has consistently shown that increased caregiver burden is associated with decreased mental and physical health.^52,61^ Family caregivers have therefore been defined as “hidden patients.”^62^ From this point of view, caregivers of cancer patients reported negative effects on their own health, such as depression, fatigue, anxiety, hopelessness, guilt, regret, fear, sleep problems, burnout, and social isolation.^63^ In addition, it has been reported that family caregivers and cancer patients experience similar levels of depression and that caregivers experience significant anxiety compared to patients.^64,65^

Considering all these, the problems experienced by health professionals should not be ignored, and consultancy services should be provided in order for caregivers to cope with the difficulties they encounter in hospital and home environments.^66,67^ Therefore, it is critical for health professionals not only to participate in the diagnosis and treatment of cancer and to solve medical problems but also to understand the spiritual needs of both individuals with cancer and their families. This support from health professionals is of great importance in the acceptance–rejection phase of the disease of patients and their relatives.^68,69^ In addition, in order to reduce the burden of caregivers and increase their self-efficacy, caregiving responsibilities should be shared with other family members and caregivers should be given more time to rest.^57,70^

## Conclusion

As a result, it is extremely important for health professionals not to ignore the problems experienced by cancer patients and caregivers while caring for cancer patients, to determine the anxiety, depression, and sleep quality levels of caregivers at regular intervals, and to prepare a holistic education plan for the problems to evaluate their implementation and results. Thus, negative effects on the psychological, social well-being, and physical of both the patient and the caregiver can be prevented.
